# The live poultry trade and the spread of highly pathogenic avian influenza: Regional differences between Europe, West Africa, and Southeast Asia

**DOI:** 10.1371/journal.pone.0208197

**Published:** 2018-12-19

**Authors:** Tong Wu, Charles Perrings

**Affiliations:** School of Life Sciences, Arizona State University, Tempe, AZ, United States of America; Nanjing Agricultural University, CHINA

## Abstract

In the past two decades, avian influenzas have posed an increasing international threat to human and livestock health. In particular, highly pathogenic avian influenza H5N1 has spread across Asia, Africa, and Europe, leading to the deaths of millions of poultry and hundreds of people. The two main means of international spread are through migratory birds and the live poultry trade. We focus on the role played by the live poultry trade in the spread of H5N1 across three regions widely infected by the disease, which also correspond to three major trade blocs: the European Union (EU), the Economic Community of West African States (ECOWAS), and the Association of Southeast Asian Nations (ASEAN). Across all three regions, we found per-capita GDP (a proxy for modernization, general biosecurity, and value-at-risk) to be risk reducing. A more specific biosecurity measure–general surveillance–was also found to be mitigating at the all-regions level. However, there were important inter-regional differences. For the EU and ASEAN, intra-bloc live poultry imports were risk reducing while extra-bloc imports were risk increasing; for ECOWAS the reverse was true. This is likely due to the fact that while the EU and ASEAN have long-standing biosecurity standards and stringent enforcement (pursuant to the World Trade Organization’s Agreement on the Application of Sanitary and Phytosanitary Measures), ECOWAS suffered from a lack of uniform standards and lax enforcement.

## 1. Introduction

Highly pathogenic avian influenzas have become a major threat to human and livestock health in the last two decades. The H5N1panzootic (2004 ongoing) has been one the most geographically widespread and costly, resulting in the loss of hundreds of millions of poultry in 68 countries [1] and over 450 human deaths worldwide—a mortality rate of 60 percent [2, 3]. For H5N1, and other H5 subtypes, most countries reporting poultry outbreaks also report evidence of the disease in wild bird populations, and the mechanisms for the spread of H5N1 have been identified as a combination of wild bird transmission and the live poultry trade [4, 5].

In this paper we reconsider the role of the poultry trade in the spread of H5N1. The background to the study is the somewhat conflicting results of earlier studies. Using phylogenetic relationships between virus isolates at the peak of the first H5N1 panzootic, Kilpatrick et al. identified pathways for 52 introductions in Asia, Europe, and Africa. They concluded that of 21 H5N1 introductions in Asia, 9 were due to the poultry trade and 3 were due to migrating birds; that of 23 introductions in Europe none were due to the poultry trade, and 20 were due to migratory birds; and that of 8 introductions in Africa, 2 were due to the poultry trade and 3 were due to migrating birds [4]. A later study of the same panzootic through 2006 found that except for introductions into northern and southern Vietnam, which were due to the poultry trade, all remaining introductions were best explained by wild bird migration. The authors argued that the spatial and temporal regularity of H5N1 introductions could only be explained by wild bird migration along four flyways: the East Asia flyway connecting the Far East of Russia, eastern China and Southeast Asia; the Black Sea-Mediterranean Sea flyway connecting Eastern Europe, the Arabian Peninsula and the Nile River Valley; the East Africa-West Asia flyway connecting western/central Siberia and Central Asia and Africa; and the Central Asia flyway connecting Siberia and northern China to the southern part of North Asia and Southwest Asia [5].

There is evidence for the transmission of a number of infectious zoonotic and epizootic diseases through commercially traded animals and animal products [6–10]. It has been shown that transmission risk increases with the volume of trade, and decreases with both the distance between source and sink areas and the biosecurity measures applied in both places [7, 8, 11]. A number of studies have identified a strongly positive relationship between the opening of new markets and the introduction of a range of animal diseases, and between growing trade volumes and the probability that those diseases will establish and spread [9, 12–26]. In the case of H5N1, phylogenetic studies have shown significant phylogenetic clustering in Southeast Asia, consistent with the high frequency circulation among the countries of that region resulting from the growth of intra-regional trade [27, 28].

The biosecurity measures applied in international trade are, in principle, regulated by the Agreement on the Application of Sanitary and Phytosanitary Measures (SPS Agreement) and the standard-setting bodies supporting that agreement (the Codex Alimentarius Commission for food products, the International Plant Protection Convention for plant products, and the World Organization for Animal Health (OIE) for animal products). Since these are not enforcement bodies, however, biosecurity standards are, in practice, regulated by bilateral and multilateral regional trade agreements [29].

**Fig 1:**
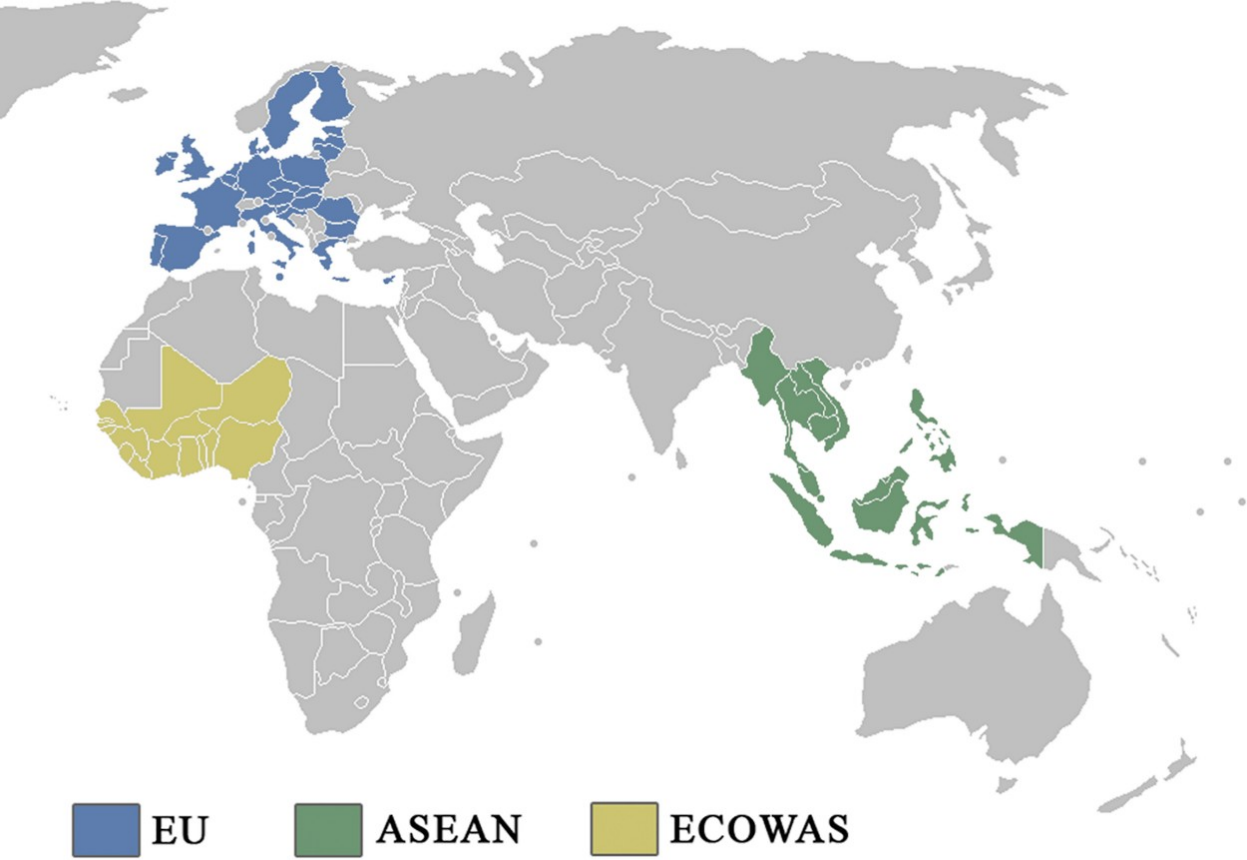
The three trade blocs heavily impacted by avian influenza under study: The European Union (EU), The Economic Community of West Africa (ECOWAS), and The Association of Southeast Asian Nations (ASEAN).

In this study, we consider transmission mechanisms in the three regions of the world where H5N1 panzootics have been most intense (as indicated by numbers of outbreaks): Europe, West Africa, and Southeast Asia. These regions are associated with three regional trade blocs: the European Union (EU), the Economic Community of West Africa (ECOWAS), and the Association of Southeast Asian Nations (ASEAN) (Fig 1). These regions are significantly different from one another in socio-economic conditions, and particularly in the biosecurity measures applied to intra-regional trade. Using data on the 12,944 H5N1 outbreaks occurring in these regions between 2004 and 2016 (Table 1), we estimate disease risk as a function of a number of risk factors, including live poultry trade volumes and various measures of trade biosecurity.

**Table 1:** The distribution of H5N1 poultry outbreaks between 2004–2016 across the member states of ASEAN, ECOWAS, and EU. “-” signifies that the country was not a member of its associated trade bloc in that given year.

	2004	2005	2006	2007	2008	2009	2010	2011	2012	2013	2014	2015	2016
*Brunei*	0	0	0	0	0	0	0	0	0	0	0	0	0
*Cambodia*	23	1	5	1	1	1	3	4	1	8	5	2	1
*Indonesia*	1	3	20	0	20	1503	1206	1155	308	260	310	107	269
*Laos*	19	0	1	7	13	6	1	0	0	0	0	0	1
*Malaysia*	0	0	5	1	0	0	0	0	0	0	0	0	0
*Myanmar*	0	0	84	15	0	0	4	9	3	0	0	4	1
*Philippines*	0	0	0	0	0	0	0	0	0	0	0	0	0
*Singapore*	0	0	0	0	0	0	0	0	0	0	0	0	0
*Thailand*	1755	194	5	8	14	1	3	1	0	0	0	0	0
*Vietnam*	2359	1631	63	259	81	58	48	47	52	7	46	20	4
***ASEAN***	**4157**	**1829**	**183**	**291**	**129**	**1569**	**1265**	**1216**	**364**	**275**	**361**	**133**	**276**
*Benin*	0	0	0	5	0	0	0	0	0	0	0	0	0
*Burkina Faso*	0	0	3	0	0	0	0	0	0	0	0	39	0
*Cabo Verde*	0	0	0	0	0	0	0	0	0	0	0	0	0
*Cote d'Ivoire*	0	0	5	0	0	0	0	0	0	0	0	29	14
*Gambia*	0	0	0	0	0	0	0	0	0	0	0	0	0
*Ghana*	0	0	0	9	0	0	0	0	0	0	0	34	27
*Guinea*	0	0	0	0	0	0	0	0	0	0	0	0	0
*Guinea-Bissau*	0	0	0	0	0	0	0	0	0	0	0	0	0
*Liberia*	0	0	0	0	0	0	0	0	0	0	0	0	0
*Mali*	0	0	0	0	0	0	0	0	0	0	0	0	0
*Niger*	0	0	4	0	0	0	0	0	0	0	0	1	2
*Nigeria*	0	0	84	63	4	0	0	0	0	0	1	254	219
*Senegal*	0	0	0	0	0	0	0	0	0	0	0	0	0
*Sierra Leone*	0	0	0	0	0	0	0	0	0	0	0	0	0
*Togo*	0	0	0	4	1	0	0	0	0	0	0	0	2
***ECOWAS***	**0**	**0**	**96**	**81**	**5**	**0**	**0**	**0**	**0**	**0**	**1**	**357**	**264**
*Austria*	0	0	4	0	0	0	0	0	0	0	0	0	0
*Belgium*	0	0	0	0	0	0	0	0	0	0	0	0	0
*Bulgaria*	-	-	-	-	0	0	0	0	0	0	0	1	0
*Croatia*	-	-	-	-	-	-	-	-	-	-	0	0	0
*Cyprus*	0	0	0	0	0	0	0	0	0	0	0	0	0
*Czech Republic*	0	0	0	3	0	0	0	0	0	0	0	0	0
*Denmark*	0	0	1	0	1	0	0	0	0	0	0	0	0
*Estonia*	0	0	0	0	0	0	0	0	0	0	0	0	0
*Finland*	0	0	0	0	0	0	0	0	0	0	0	0	0
*France*	0	0	1	0	0	0	0	0	0	0	0	15	6
*Germany*	0	0	5	9	1	0	0	0	0	0	0	0	0
*Greece*	0	0	0	0	0	0	0	0	0	0	0	0	0
*Hungary*	0	0	27	2	0	0	0	0	0	0	0	0	0
*Ireland*	0	0	0	0	0	0	0	0	0	0	0	0	0
*Italy*	0	0	0	0	0	0	0	0	0	0	0	0	0
*Latvia*	0	0	0	0	0	0	0	0	0	0	0	0	0
*Lithuania*	0	0	0	0	0	0	0	0	0	0	0	0	0
*Luxembourg*	0	0	0	0	0	0	0	0	0	0	0	0	0
*Malta*	0	0	0	0	0	0	0	0	0	0	0	0	0
*Netherlands*	0	0	0	0	0	0	0	0	0	0	0	0	0
*Poland*	0	0	0	8	0	0	0	0	0	0	0	0	0
*Portugal*	0	0	0	0	0	0	0	0	0	0	0	0	0
*Romania*	-	-	-	-	0	0	2	0	0	0	0	0	0
*Slovakia*	0	0	0	0	0	0	0	0	0	0	0	0	0
*Slovenia*	0	0	0	0	0	0	0	0	0	0	0	0	0
*Spain*	0	0	0	0	0	0	0	0	0	0	0	0	0
*Sweden*	0	0	1	0	1	0	0	0	0	0	0	0	0
*United Kingdom*	0	0	0	3	1	0	0	0	0	0	0	0	0
***EU***	**0**	**0**	**39**	**25**	**4**	**0**	**2**	**0**	**0**	**0**	**0**	**16**	**6**

Source: Emergency Prevention System for Animal Health (http://empres-i.fao.org/eipws3g).

## 2. Materials and methods

We follow Kilpatrick, Chmura (4) in considering the relative risks posed by migratory birds and trade in the spread of H5N1, but with an additional decade of data on both outbreaks and risk factors. Our primary interest is in the role of live poultry imports as a source of trade-related avian influenza risk at the regional level. We note that other poultry products, such as packaged meat and eggs, do pose a risk, but it is significantly lower. Although avian influenza can persist in frozen meat, contact with that meat is unlikely to cause infection [30]. Furthermore, since HPAIs are lethal to egg embryos, eggs are not a potential source of transmission [31]. The data comprise an unbalanced panel covering 53 countries over 13 years; the lack of balance is due to the fact that membership of the EU changed over the timeframe.

The response variable in all models estimated was a log transformation of the number of H5N1 poultry outbreaks in a given country in a given year, obtained from the Emergency Prevention System for Animal Health (EMPRES), a joint project of the FAO and OIE [32]. The log transformation was applied to account for the wide disparities in the numbers of the outbreaks across countries. In 2010, for example, Indonesia recorded 1206 outbreaks while Romania, the only EU country to be infected that year, had only 2. In addition to reflecting the differing directions and intensities of risk factors, this also reflects differences in reporting conventions for H5N1 at the international level [33]. A series of outbreaks may be reported separately in one country, but be treated as a single event in another.

Data on trade in live poultry were obtained from the United Nations’ Comtrade Database (comtrade.un.org) and resourcetrade.earth, a project of the Royal Institute of International Affairs (www.chathamhouse.org). These report the total imports of live poultry into a given country in a given year by weight (kg). The data on trade in live poultry did not distinguish between different types of domestic birds, such as chickens, duck, and geese, but grouped them under a single commodity category of “live poultry.”

With respect to wild bird migration as a pathway for H5N1 spread, we used the density of wild bird habitat as a proxy for the presence and scale of migratory bird populations, and the likelihood that wild and domestic birds will mix. Lakes, wetlands, and (irrigated) agricultural areas have been consistently identified as wintering and breeding grounds for migratory birds, and as places where wild birds may come into contact with free-ranging poultry [34–41]. The indicator for wild bird habitat used in this study was the set of “Important Bird and Biodiversity Areas” (IBAs) for “migratory and congregatory waterbirds” identified by BirdLife International (datazone.birdlife.org). In their 2006 analysis of H5N1 spread, Kilpatrick, Chmura (4) also identified IBAs as a proxy for migratory birds and the infection risks they pose.

Country-level statistics on socioeconomic and agro-ecological conditions were taken from the United Nations’ Food and Agriculture Organization (www.fao.org/faostat/en/) and the World Bank (data.worldbank.org). Agricultural land cover was reported as a percentage of total land area of the country. Per-capita GDP was reported in purchasing power parity terms as current international dollars. Data for 2016 for these two variables were missing for certain countries. In these cases, the gaps were filled by extrapolating the missing data as a linear trend of the preceding 11 years. We assume that agricultural land–where free-ranging chickens, ducks, and geese are commonly raised in all three regions–also acts as a relevant proxy for susceptible poultry.

Data on the biosecurity measures targeting avian influenza undertaken by each country were obtained from the World Organisation for Animal Health (OIE) (www.oie.int). These report a standardized series of biosecurity controls targeting wildlife and livestock diseases, including those related to surveillance, vaccination, border checks, and management of wild disease reservoirs, and whether or not a given country undertook them in a given year. We chose a subset of these biosecurity measures we considered most relevant to H5N1 avian influenza risks for inclusion in our model. Additionally, in any given year, there were 1 to 4 countries that did not provide a report of biosecurity measures to the OIE; we assumed that this indicates an absence of action, and the dataset records these cases as zeroes.

Our modeling approach relied on generalized linear models (GLM) to analyze a panel of data on disease outbreaks and associated risk factors. In this we follow others who have sought to predict the spread of H5N1 at both national and international levels [42–44] or H7N9 [45–47]. GLMs are well suited to epidemiological studies because of their flexibility regarding data type and the distribution of response variables, their simplicity of application, and their frequency of use [33].

Our identification strategy involved the selection of three specifications for each of two estimators. We adopted both random and fixed effects estimators. Hausman tests conducted at the all-regions level favored a random effects estimator, as the *p-*value exceeded the 5% threshold below which fixed-effects regression is conventionally considered necessary. Some factors that influence the likelihood and number of outbreaks in a given country or region are not likely to change significantly over the course of several years, or even a decade. In our dataset, for example, the amount of land covered by wild bird habitat is time-variant, while agricultural land and even per-capita GDP for many countries experienced relatively modest variations over the timeframe of the study. In this case, and as the Hausman diagnostics indicate, a random effects estimator is more appropriate. Nevertheless, since we wished to control for time-invariant characteristics of regions and countries we also implemented fixed effects estimators at both the aggregate and trading bloc levels, implicitly assuming no changes in the trade or biosecurity environment at the bloc level that we are unable to control for.

Our first specification (Model 1) included a number of factors related to disease risk but excluded both live poultry imports and biosecurity measures. Included predictors were land area, human population, per-capita GDP in purchasing power terms, agricultural area, wild bird habitat area, and the live chicken population. Our second specification (Model 2) added intra-regional trade bloc and extra-bloc imports of live poultry. Our third specification (Model 3) added four main biosecurity measures: border precautions, general surveillance, vaccination prohibition, and wild disease reservoir management. All are categories of OIE-reported biosecurity measures taken against avian influenza.

The general forms of the estimated random and fixed effects models were:

$$y_{it}=\beta_{0}+\overset{6}{\underset{j=1}{\sum }}X_{jit}\beta_{j}+\overset{2}{\underset{k=1}{\sum }}Z_{kit}\beta_{k}+\overset{4}{\underset{s=1}{\sum }}U_{sit}\beta_{s}+\delta_{1}ecowas+\delta_{2}asean+u_{i}+\varepsilon_{it},$$ (1)

$$y_{it}=\beta_{0}+\overset{6}{\underset{j=1}{\sum }}X_{jit}\beta_{j}+\overset{2}{\underset{k=1}{\sum }}Z_{kit}\beta_{k}+\overset{4}{\underset{s=1}{\sum }}U_{sit}\beta_{s}+u_{i},$$ (2)

where *y*_*it*_ denotes the number of poultry outbreaks in country *i* in year *t*, *X* includes the predictors for Model 1, *Z* includes the additional predictors for Model 2, *U* includes the additional predictors for Model 3, *ecowas and asean* are dummy variables for the two titular regional trade blocs (the EU is the reference group), and *u*_*it*_ and *ε_it_* are the “between” and “within” errors respectively. To account for heteroskedasticity, we used robust standard errors. Finally, since the data used in this analysis are reported annually, and H5N1 has been a conspicuous and fast-moving epidemic (meaning the effects of an outbreak are unlikely to persist over a long period of time) among poultry, we did not use a lag structure in our statistical analysis. Therefore, we assumed that the factors driving an outbreak in a given year are contemporaneous with it (e.g., an outbreak that occurred in 2012 were modelled using trade volumes from 2012). We were also constrained by data availability in our use of annual increments: although monthly data exist for outbreaks, they do not for important predictor variables such as per-capita GDP, human and poultry populations, the volume of live poultry traded, and biosecurity.

## 3. Results

Regressions results from all models, including both random and fixed effects, are reported in Tables 2–5. At the all-regions level, the results for the random- and fixed-effects models were very similar, with the same set of predictor variables being statistically significant (i.e., *p-*values below the 5% or 10%) and the same direction of impact on the response variable. This set of predictors was human population (positive direction), per-capita GDP (negative direction), intra-trade bloc live poultry imports (negative direction), extra-trade bloc live poultry imports (positive direction), and the biosecurity measure of surveillance (negative direction).

**Table 2:** Results from the regression models of H5N1 outbreak risk factors for member states in all three regions; regressor coefficients are reported and statistically-significant factors are marked by asterisks. A blank space signifies that the variable was not included in the given model.

Variables	Units	Model 1		Model 2		Model 3	
		Random Effects	Fixed Effects	Random Effects	Fixed Effects	Random Effects	Fixed Effects
Population	# people	1.07x10^-8^**	4.51x10^-8^**	1.18x10^-8^**	4.47x10^-8^**	1.20x10^-8^ **	4.51x10^-8^**
Area	km^2^	-2.80x10^-7^	-0.0000225	-3.40x10^-7^	-8.41x10^-6^	-3.45x10^-7^	-2.94x10^-6^
Agricultural land	% land area	-0.00218	-0.0106	-0.00353	-0.00691	-0.00387	-0.00923
Live chickens	1000 birds	1.27x10^-6^	4.75x10^-7^	1.26x10^-6^	5.42x10^-7^	1.24x10^-6^	4.22x10^-7^
IBA for waterbirds	% land area	0.0000649	-0.176	0.0000390	-0.164*	0.0000872	-0.154**
Per-capita GDP	current international $	-9.16x10^-6^ **	-0.0000171**	-7.80x10^-6^*	-0.0000138**	-5.99x10^-6^	-0.0000108*
Intra-bloc live poultry imports	kg			-5.57x10^-6^**	-4.82x10^-6^**	-4.87x10^-6^ **	-3.14x10^-6^**
Extra-bloc live poultry imports	kg			0.00174v**	0.00189**	0.00171**	0.00184**
Border precautions	presence/absence					0.130	0.196
General surveillance	presence/absence					-0.304*	-0.370*
Vaccination prohibited	presence/absence					0.0675	0.122
Wild reservoirs management	presence/absence					0.172	0.175
ECOWAS	*dummy*	0.449		0.327		0.415	
ASEAN	*dummy*	-0.0195		0.00850		0.164	
Observations		621	621	621	621	621	621
Within R^2^		0.0303	0.0410	0.0673	0.0785	0.0959	0.1082
Between R^2^		0.7239	0.0114	0.7159	0.0072	0.7036	0.0277
Overall R^2^		0.4496	0.0063	0.4599	0.0049	0.4638	0.0181
Rho		0.2764	0.9978	0.2940	0.9974	0.3159	0.9971

** statistically significant at the 5% level

* statistically significant at the 10% level

**Table 3:** Results from the regression models of H5N1 poultry outbreak risk factors for the Association of Southeast Asian Nations (ASEAN); regressor coefficients are reported and statistically-significant factors are marked by. A blank space signifies that the variable was not included in the given model.

Variables	Units	Model 1		Model 2		Model 3	
		Random Effects	Fixed Effects	Random Effects	Fixed Effects	Random Effects	Fixed Effects
*Population*	# people	6.20x10^-8^**	1.92x10^-7^	8.96x10^-8^**	1.65x10^-7^	1.13x10^-8^	1.83x10-7
*Area*	km^2^	-5.96x10^-6^*	0.00353**	-8.29x10^-6^	0.00417**	-1.13x10^-6^	0.00445**
*Agricultural land*	% land area	-0.174**	-0.647**	-0.244**	-0.549**	-0.0467**	-0.593**
*Live chickens*	1000 birds	9.85x10^-7^	-1.02x10^-6^	4.08x10^-7^	-4.59x10^-7^	1.69x10^-6^	-1.18x10-6
*IBA for waterbirds*	% land area	-0.0290	0.322	-0.0577	0.190	-0.0122	0.380
*Per-capita GDP*	current international $	-0.0000522*	-7.25x10^-6^	-0.0000447*	-8.48x10^-6^	-0.0000317**	-8.33x10-7
*Intra-bloc live poultry imports*	kg			-4.86x10^-6^	1.00x10^-6^	-0.0000108	0.0000112
*Extra-bloc live poultry imports*	kg			0.00212**	0.00171**	0.00222**	0.00187**
*Border precautions*	presence/absence					0.398	1.23**
*General surveillance*	presence/absence					-0.625	-0.606*
*Vaccination prohibited*	presence/absence					-1.00*	-0.267
*Wild reservoirs management*	presence/absence					0.881**	-0.533*
*Observations*		120	120	120	120	120	120
*Within R*^*2*^		0.1626	0.3038	0.2596	0.3472	0.2036	0.4759
*Between R*^*2*^		0.4346	0.5503	0.2800	0.5504	0.8057	0.5503
*Overall R*^*2*^		0.3252	0.3674	0.2346	0.3674	0.5921	0.3674
*Rho*		0.7242	1.000	0.8798	1.000	0.0000	1.000

** statistically significant at the 5% level

* statistically significant at the 10% level

**Table 4:** Results from the regression models of H5N1 poultry outbreak risk factors for the Economic Community of West African States (ECOWAS); regressor coefficients are reported and statistically-significant factors are marked by asterisks. A blank space signifies that the variable was not included in the given model.

Variables	Units	Model 1		Model 2		Model 3	
		Random Effects	Fixed Effects	Random Effects	Fixed Effects	Random Effects	Fixed Effects
Population	# people	6.05x10^-9^	3.85x10^-8^**	7.34x10^-9^	4.21x10^-8^**	1.82x10^-9^	1.95x10-8*
Area	km^2^	1.30x10^-8^	0	-1.40x10^-8^	0	-3.50x10^-8^	0
Agricultural land	% land area	0.00247	0.00434	0.00233	0.00907	-0.00461	0.00626
Live chickens	1000 birds	2.02x10^-7^	0.0000112	-3.24x10^-7^	0.0000113	3.12x10^-6^	9.58x10-6
IBA for waterbirds	% land area	-0.00744*	0	-0.00646	0	-0.0119**	0
Per-capita GDP	current international $	0.000198*	0.0000887	0.000173	0.000172	0.000290**	0.000303
Intra-bloc live poultry imports	kg			0.0122**	0.0124**	0.0138**	0.0107**
Extra-bloc live poultry imports	kg			-0.000638	-0.000990**	-0.000895*	-0.000815
Border precautions	presence/absence					-0.552**	-0.741
General surveillance	presence/absence					0.103	0.0430
Vaccination prohibited	presence/absence					0.476**	0.540
Wild reservoirs management	presence/absence					0.477**	0.266
Observations		180	180	180	180	180	180
Within R^2^		0.0477	0.0582	0.0603	0.0782	0.1506	0.1722
Between R^2^		0.8843	0.8672	0.8895	0.8733	0.8752	0.8314
Overall R^2^		0.2492	0.2354	0.2648	0.2420	0.3303	0.2786
Rho		0.0000	0.7844	0.0000	0.8101	0.0000	0.6304

** statistically significant at the 5% level

* statistically significant at the 10% level

**Table 5:** Results from the regression models of H5N1 poultry outbreak risk factors for the European Union (EU); regressor coefficients are reported and statistically-significant factors are marked by asterisks. A blank space signifies that the variable was not included in the given model.

Variables	Units	Model 1		Model 2		Model 3	
		Random Effects	Fixed Effects	Random Effects	Fixed Effects	Random Effects	Fixed Effects
Population	# people	6.34x10^-9^**	1.37x10^-7^	9.46x10^-9^*^a^	1.09x10^-7^	1.07x10^-8^**	1.29x10-7
Area	km^2^	3.88x10^-7^	-0.00001	2.65x10^-7^	-8.15x10^-6^	2.79x10^-7^	-0.0000149
Agricultural land	% land area	0.00333^a^	0.0410**	0.00293	0.0395^a^	0.00311	0.0399
Live chickens	1000 birds	-2.47x10^-6^**	-8.66x10^-6^^a^	-2.98x10^-6^**	-8.03x10^-6^	-3.51x10^-6^**	-8.58x10-6
IBA for waterbirds	% land area	0.0000163	-0.109**	4.30x10^-6^	-0.115**	0.000116	-0.103**
Per-capita GDP	current international $	-1.74x10^-6^	-9.03x10^-6^^a^	-1.51x10^-6^	-7.34x10^-6^	-3.04x10^-7^	-9.52x10-6^a^
Intra-bloc live poultry imports	kg			-2.82x10^-6^	-3.42x10^-6^**	-3.34x10^-6^^a^	-2.22x10-6
Extra-bloc live poultry imports	kg			-0.00413	-0.00517	-0.00471	-0.00459
Border precautions	presence/absence					0.0377	-0.116
General surveillance	presence/absence					-0.0476	-0.0706
Vaccination prohibited	presence/absence					0.107**	0.188**
Wild reservoirs management	presence/absence					0.0657	0.0812
Observations		321	321	321	321	321	321
Within R^2^		0.0835	0.1134	0.0986	0.1319	0.0970	0.1552
Between R^2^		0.3471	0.0384	0.2584	0.0319	0.3218	0.0271
Overall R^2^		0.0682	0.0051	0.0858	0.0043	0.1075	0.0038
Rho		0.0079	0.9992	0.0045	0.9993	0.0187	0.9992

** statistically significant at the 5% level

* statistically significant at the 10% level

Additionally, although the coefficient values for the same predictor differed between the two estimators, all pairs were within the same order of magnitude. The only exception to this was migratory waterbird habitat variable—the percent of land area covered by IBAs for migratory and congregatory waterbirds. This was statistically significant and negative (i.e., had a mitigating impact on H5N1 poultry outbreaks) for the fixed-effects model but was not significant for the random-effects model. The overall R-squared for the random-effects model was significantly higher than that for the fixed-effects model (0.451 vs. 0.0181). The “between R-squared” value was particularly high (0.682) in the random effects model, signaling the importance of variation among countries (as opposed to “within R-squared,” which measures the variation within countries over time).

As we had expected, we found significant differences across trade regions. In the random-effects model, ECOWAS diverged from all-regions conditions and from ASEAN with respect to per-capita GDP and extra-bloc imports: while the two predictors were, respectively, risk-decreasing and risk-increasing at the all-regions level and in ASEAN, they had the opposite impacts in ECOWAS. Furthermore, ECOWAS differed from the all-regions level and from the EU in terms of intra-bloc imports: while this was risk-decreasing for the former two, it was risk-increasing for ECOWAS.

Finally, there were predictors that were statistically insignificant at the all-regions level but had a significant effect within different regions. For ASEAN, agricultural land cover was a mitigating factor for outbreaks while wild disease reservoir management showed a strong positive relation with outbreaks. For ECOWAS, wild waterbird habitats and border precautions had a mitigating effect on outbreaks while vaccination prohibition and wild reservoir management had a positive effect. In the EU, the population of live chickens had a strong negative relation with outbreaks, while vaccination prohibition, similar to the case with ECOWAS, was positively related.

## 4. Discussion

Following Liang, Xu (5), there is a perception that the long distance transmission of highly pathogenic avian influenza H5N1 was largely due to wild bird migration, with the live poultry trade playing a minor and more localized role in some cases. Our concern here has been to identify the nature of the risk posed by the live poultry trade in different regions of the world, and the conditions affecting that risk.

Our measure of development status, per-capita GDP, is simultaneously a proxy for modernization, biosecurity, consumption, and value-at-risk. As a proxy for modernization, it reflects risk-reducing differences in production methods. Industrial livestock production methods typically include on-farm biosecurity measures that protect poultry from contact with disease-carrying wild birds. Unlike traditional methods of free-range or “backyard” husbandry, factory production minimizes the likelihood of poultry intermingling with wild birds or being exposed to environmental pathogen pollution. For all its epidemiological, ecological, and ethical problems, industrial livestock production allows for more timely and widespread disease surveillance and vaccination, and for greater compliance with animal health regulations [48].

At the same time, per-capita GDP growth is also associated with risk-increasing changes in meat consumption, and hence poultry production. Indeed, the highest income elasticity of demand for meat and fish has been found in the poorest households and the poorest countries [49]. In developing countries, 71% of the additions to meat consumption are from pork and poultry, with poultry dominating pork [50]. Absent changes in on-farm biosecurity, increased production implies increased risk. Across all regions, the net effect of income growth is to reduce risk, dominating risk-increasing changes. In the ECOWAS region—the lowest income region—the effect is the opposite. The risk-increasing effects of income growth dominate the risk reducing effects (Table 3).

Amongst the landscape variables—land area, the proportion in agriculture, and the proportion in IBAs—our results reveal no uniform relation to H5N1 outbreaks. At the all-regions level we found a weakly negative relation between outbreaks and the proportion of the land area in IBAs (Table 1). This was driven by the European Union, which includes the highest proportion of land area in IBAs, but also the most industrialized forms of poultry production. The degree to which poultry production is industrialized also shows up in the coefficients on poultry numbers, which are negative and significant only for the EU (Table 4). While spatial heterogeneity at the landscape scale is important in terms of avian ecology, we were unable to take explicit account of these more detailed considerations in a country-scale analysis. The impacts of regional differences in biophysical conditions that are not directly controlled for are, however, included in bloc-level fixed effects.

Our primary concern is with the role of the live poultry trade, and how that differs between regions. Across all regions we find that live poultry imports into a trade bloc are risk increasing. This is consistent with past studies that have shown that extra-bloc live poultry imports may be a significant source of additional avian influenza risk where they do not meet bloc sanitary and phytosanitary standards. The EU’s common market and the ASEAN free trade regime in particular have long-standing and standardized protocols, in accordance with the World Trade Organization’s Agreement on the Application of Sanitary and Phytosanitary Measures. But the two blocs have quite different exposures to external risk. A study of highly pathogenic avian influenza introductions to Vietnam, for example, found that extra-ASEAN imports of live poultry increased the risk of introduction [51]. This is also what our study finds for the ASEAN region (Table 2). We do not see an equivalent effect for the EU (Table 4), reflecting differences in both import volumes and the biosecurity measures applied to imports. The EU imports less and applies stricter biosecurity measures to those imports. The ECOWAS story is different. Extra-bloc live poultry imports are risk reducing, not risk increasing (Table 3). It is likely that imports from outside the bloc reduce avian influenza risk in the region in part because they meet biosecurity standards that are more stringent than the standards applied in the region.

The effects of intra-bloc trade in live poultry mirror the effects of extra-bloc trade. In the EU and ASEAN, intra-bloc trade is risk reducing (Tables 2 and 4). This may reflect a “substitution effect” in which imports of safer intra-bloc poultry crowds out riskier extra-bloc imports. Other studies have come to similar conclusions. EU-derived live poultry imports to Spain, for example, were found to pose no threat of avian influenza introduction [52]. Once again, ECOWAS is the exception. Extra-ECOWAS imports of live poultry are risk reducing while intra-bloc imports are risk increasing (Table 2). This is likely due to poor internal biosecurity, such as lax standards and inconsistent execution of inspections. Regulatory standards within the ECOWAS trade bloc have been weak for the whole of the study period [53]. While harmonized sanitary and phytosanitary standards for the 15 member states of ECOWAS were in principle adopted in 2010, most ECOWAS states had yet to submit legislation for international certification by 2017 [54].

Failure to adopt and enforce unified standards may be partly due to income constraints in ECOWAS countries. In PPP terms, the bloc’s per-capita GDP in 2016 was less than half that of ASEAN and approximately 1/8^th^ that of the EU, meaning it had less resources available for biosecurity policies and institutions. Political instability may be another important obstacle: a number of ECOWAS member states, including Nigeria, Niger, Sierra Leone, Mali, Liberia, and Cote d’Ivoire have suffered from civil wars and armed insurgencies over the past two decades. Such fraught geopolitical conditions are not conducive to the establishment and enforcement of cross-border regulations. It goes without saying, though, that certification of sanitary and phytosanitary legislation in ECOWAS states, and the establishment of enforcement agencies to bring states into compliance with the SPS Agreement and Codex Alimentarius is a necessary condition of improving regional trade-related biosecurity.

In terms of biosecurity measures more specifically, we did not have direct measures of on-farm biosecurity (but conjecture that biosecurity is increasing in per-capita GDP), but we did have measures of four biosecurity policies at the national level. These include: (1) border precautions (measures applied at airports, ports, railway stations or road check-points open to international movement of animal, animal products and other related commodities, where import inspections are performed to prevent the introduction of the disease, infection or infestation); (2) general surveillance (surveillance not targeted at a specific disease, infection or infestation); (3) prohibition of vaccination (prohibition of the use of a vaccine to control or prevent the infection or infestation); and (4) management of wildlife reservoirs (measures to reduce the potential for wildlife to transmit the disease to domestic animals and human beings). The management of wild disease reservoirs differs widely across countries, but techniques include vaccination, treatment of infections with drugs, isolation of infected populations, population translocation, reproduction reduction, culling, and control (draining, flooding, or burning) of wild disease reservoir habitat [55].

Of these measures, only general surveillance was significant at the all-regions level, while at the bloc level the effects of the different measures were frequently ambiguous. In the EU, for example, only the prohibition of vaccination was significant, and then in positive relation to outbreaks. For poultry, vaccination may be prohibited because the practice makes it difficult to distinguish infected from vaccinated flocks. This makes it a concomitant of policies centered on livestock culling as the primary response to outbreak risk [56]. No other biosecurity policy was found to have a statistically significant relation to outbreaks in the region.

The same set of policies had opposite effects in ASEAN and ECOWAS. The prohibition of vaccination and the management of wild reservoirs were positively related to outbreaks in ECOWAS but negatively related to outbreaks in ASEAN, while border protection measures were negatively related to outbreaks in ECOWAS but positively related to outbreaks in ASEAN. This may reflect regional disparities in the quality of implementation not captured in the data. But it may also reflect the greater importance of trade in the transmission of the disease in ECOWAS.

In their survey of the international spread of H5N1 in the early years of the global epidemic, Kilpatrick, Chmura (4) found that transmission into Europe was by wild birds, that transmission into Southeast Asia was by the poultry trade, and transmission into Africa by a balance of both. Our results suggest that after introduction, inter-country spread had differing dynamics in each region. While intra-bloc trade facilitated H5N1 spread among West African countries, it did not in either Europe or Southeast Asia. In these areas, greater risk was posed by out-of-region live poultry imports.

## 5. Conclusion

In recent decades, avian influenzas have emerged as a major threat to human and animal health across the world. In particular, HPAI H5N1, which was first isolated in 1996, has been the most widespread and among the most devastating in terms of livestock and human mortality. It has inflicted severe losses to poultry stocks and caused hundreds of human deaths. Even today, as other avian influenzas have become epidemic, H5N1 remains in circulation among wildlife and livestock. Identifying and quantifying the mechanisms of its international spread can help lay the groundwork for prediction and mitigation. It may also provide an instructive framework for the management of other avian influenzas.

In this study, we considered the risk posed by the international trade in live poultry and the effects of associated biosecurity measures. Differing agro-ecological and socioeconomic conditions across the trade regions were shown to influence epidemic dynamics in different ways, with certain factors being risk-enhancing or risk-decreasing in one region but having the opposite effect, or no significant effect, in another. In policy terms, there is no one-size-fits-all solution to mitigating avian influenza spread. The particular conditions, including those related to the trade agreements and associated regulatory standards, of a given region need to be carefully considered. But overall, biosecurity measures are potentially effective at controlling H5N1 risks, and should be undertaken as a means to forestall spread–in general, mitigation of epidemics is significantly more cost-efficient than suppression [57]. On-farm and other forms of domestic biosecurity may be more important than trade-related measures, but where the protection of trade pathways is weak, the risk of avian influenza spread is clearly higher.

## Supporting information

S1 File Detailed information on data sources. The public sources of the data used in this study, and how they were acquired, are described.
(DOCX)
