# Supplementary material for: The live poultry trade and the spread of highly pathogenic avian influenza: Regional differences between Europe, West Africa, and Southeast Asia
Source: PLoS One. 2018 Dec 19;13(12):e0208197. doi: 10.1371/journal.pone.0208197 (PMC6300203; doi:10.1371/journal.pone.0208197)
Supplement: S1 File — The public sources of the data used in this study, and how they were acquired, are described. (DOCX) [file pone.0208197.s001.docx]

**Supporting Information 1: Detailed information on data sources**

Data used in our analysis are all publicly available. Data on H5N1 poultry outbreaks were obtained from the Emergency Prevention System for Animal Health (<http://empres-i.fao.org/eipws3g/>). Using the “advanced search” feature, we searched for HPAI H5N1 outbreaks in domesticated animals occurring between 1 January 2004 and 31 December 2016 for the 53 countries that comprise the Association of Southeast Asian Nations (10 member states), the Economic Community of West African States (15 member states), and the European Union (28 member states).

Data on the live poultry trade were downloaded from “resourcetrade.earth” (<https://resourcetrade.earth/>), a project of the Royal Institute of International Affairs (<http://www.chathamhouse.org>), and based on data from the United Nations’ Comtrade Database (<http://comtrade.un.org/>). We searched for the quantity of “live poultry” imports and exports (in kg) for each of the 53 countries listed above for the years 2004-2016.

Data on the area of migratory waterbird habitat for each of the 53 countries were downloaded from the BirdLife International database (<http://datazone.birdlife.org>). We queried the amount of area in each country that qualified as “Important Bird and Biodiversity Area (IBA) classification A4”, which refers to important habitats for migratory waterbird congregations.

Data on the annual agricultural land cover and live chicken stock of each of the 53 countries were downloaded from the United Nations’ Food and Agriculture Organization (<http://www.fao.org/faostat/en/>). In the FAOSTAT database, data for agricultural land cover were in the “land cover” category of “Agri-Environmental Indicators”; data for live chicken stock were in the “live animals” category of “Production”.

Data on annual per-capita GDP of each of the 53 countries were downloaded from the World Bank database (<http://data.worldbank.org>), using the search term/indicator name “GDP per capita, PPP (current international $)”.

Data for the biosecurity measures each of the 53 countries undertook in the years 2004-2016 were taken from the World Organisation for Animal Health (OIE) (<http://www.oie.int>/). In the World Animal Health Information Database (WAHIS) (<http://www.oie.int/wahis_2/public/wahid.php/Wahidhome/Home/>), we used the “Disease control measures” dataset of the “Control measures” section. In the search interface, we identified “Highly pathogenic avian influenza” as the targeted disease, and, identifying each country and year, generated a report indicating the biosecurity measures against said disease said country undertook in said year.
